# Anticancer Activity of Sulforaphane: The Epigenetic Mechanisms and the Nrf2 Signaling Pathway

**DOI:** 10.1155/2018/5438179

**Published:** 2018-06-06

**Authors:** Xuling Su, Xin Jiang, Lingbin Meng, Xiaoming Dong, Yanjun Shen, Ying Xin

**Affiliations:** ^1^Key Laboratory of Pathobiology, Ministry of Education, Jilin University, Changchun, China; ^2^Department of Radiation Oncology, The First Hospital of Jilin University, Changchun, China; ^3^Department of Internal Medicine, Florida Hospital, Orlando, FL, USA; ^4^Department of Pathology, Shanxi Medical University, Taiyuan, China

## Abstract

Sulforaphane (SFN), a compound derived from cruciferous vegetables that has been shown to be safe and nontoxic, with minimal/no side effects, has been extensively studied due to its numerous bioactivities, such as anticancer and antioxidant activities. SFN exerts its anticancer effects by modulating key signaling pathways and genes involved in the induction of apoptosis, cell cycle arrest, and inhibition of angiogenesis. SFN also upregulates a series of cytoprotective genes by activating nuclear factor erythroid-2- (NF-E2-) related factor 2 (Nrf2), a critical transcription factor activated in response to oxidative stress; Nrf2 activation is also involved in the cancer-preventive effects of SFN. Accumulating evidence supports that epigenetic modification is an important factor in carcinogenesis and cancer progression, as epigenetic alterations often contribute to the inhibition of tumor-suppressor genes and the activation of oncogenes, which enables cells to acquire cancer-promoting properties. Studies on the mechanisms underlying the anticancer effects of SFN have shown that SFN can reverse such epigenetic alterations in cancers by targeting DNA methyltransferases (DNMTs), histone deacetyltransferases (HDACs), and noncoding RNAs. Therefore, in this review, we will discuss the anticancer activities of SFN and its mechanisms, with a particular emphasis on epigenetic modifications, including epigenetic reactivation of Nrf2.

## 1. Introduction

Numerous studies have suggested that high dietary intake of cruciferous vegetables is correlated with a low risk of cancer [1]. The anticancer activity of cruciferous vegetables has been mainly attributed to isothiocyanates, which are a product of myrosinase-mediated glucosinolate degradation. Sulforaphane (SFN) is a naturally occurring isothiocyanate derived from the consumption of cruciferous vegetables, such as broccoli, cabbage, and kale. Because of its efficacy, safety, nontoxicity, lack of side effects, and low cost, bioactive SFN is widely recognized as a promising chemopreventive agent with effects against many kinds of cancers, such as cervical [2], breast [3], and bladder cancer [4]; renal cell carcinoma (RCC) [5]; non-small-cell lung cancer (NSCLC) [6]; and colon and prostate cancers [7]. SFN has also been reported to improve the efficacy of low-dose cisplatin (CDDP), a commonly used chemotherapeutic drug [8].

Studies on the mechanisms underlying the anticancer activities of SFN indicate that its regulatory effects on the tumor cell cycle, apoptosis, and angiogenesis are mediated by modulation of the related signaling pathways and genes. Cell cycle analysis showed that SFN caused G2/M phase arrest leading to inhibition of tumor proliferation/growth, which was associated with downregulation of cyclin B1 [2] and cyclin D1 genes [9], as well as increased protein levels of p21^WAF1/CIP1^ (an inhibitor of cyclin-dependent kinases) [9]. SFN also increased the expression of the proapoptotic protein Bax and decreased expression of the antiapoptotic protein Bcl-x to induce apoptosis in cancer cells [10]. By suppressing the expression and activity of hypoxia inducible factor-1*α* (HIF-1*α*) and vascular endothelial growth factor (VEGF), SFN inhibited the angiogenesis and metastasis of ovarian and colon cancers [11, 12].

SFN was also reported to be a strong activator of nuclear factor erythroid-2 (NF-E2-) related factor 2 (Nrf2). It is well known that long-term exposure to oxidative stress is an important carcinogenesis-promoting factor that induces DNA damage, mutations, and inflammation [13]. Nrf2 is a critical transcription factor in the antioxidant stress response. Activation of Nrf2 by SFN induced the expression of a battery of cytoprotective genes with anticarcinogenesis activities [14–16]. Those Nrf2-mediated cytoprotective genes include the antioxidants and phase II enzymes, such as NAD(P)H:quinone oxidoreductase-1 (NQO1), heme oxygenase 1 (HO-1), catalase, glutamate-cysteine ligase (GCL), glutathione S transferase (GST), UDP-glucuronosyltransferases (UGT), epoxide hydrolase, and superoxide dismutase (SOD). A number of studies revealed that the effects of SFN on Nrf2 and its downstream cytoprotective genes are through modification of Keap1 cysteine residues [17]; activation of mitogen-activated protein kinase (MAPK), phosphatidylinositol 3-kinase (PI3K), and protein kinase C (PKC) pathways; and epigenetic modifications, which resulted in the phosphorylation, nuclear accumulation, and increased transcription and stability of Nrf2 [18–21].

In recent years, the epigenetic mechanisms underlying the anticancer effects of SFN have received increasing attention [22]. Epigenetic modification refers to the heritable changes in gene expression that do not affect the DNA sequence itself. In mammals, epigenetic modifications mainly include DNA methylation, histone modifications (acetylation, phosphorylation, and methylation), and noncoding RNA regulation. Epigenetic changes are reversible and can readily respond to natural bioactive dietary compounds [23], such as SFN. SFN was shown to regulate the gene activation or silencing involved in cancer through epigenetic modifications [22]. Therefore, in this review, we present the anticancer activities of SFN and its epigenetic mechanisms, including epigenetic reactivation of Nrf2. This information will help facilitate the discovery and development of novel anticancer drugs.

## 2. Epigenetics and Cancer

In the classic view, cancer results from genetic alterations including mutations, insertions, deletions, copy number gains, recombination, genomic instability, and single-nucleotide polymorphisms (SNPs) [24, 25]. The mutations of tumor suppressor genes and/or oncogenes contribute to the loss of normal function or gain of abnormal expression in cancers. For example, mutations of tumor suppressors of P53 and PTEN (phosphatase and tensin homolog deleted on chromosome ten) or BRCA 1/2 (crucial proteins involved in homologous recombination) were associated with colorectal [26–28], breast, and ovarian cancer [29, 30]. In addition, TP53 and CTNNB1 (encoding *β*-catenin) exhibited point mutations and small deletions in hepatocellular carcinoma [31]. However, emerging evidence indicates that cancer can occur without a change in the nucleotide sequence, through so-called epigenetic alterations. In fact, a combinational crosstalk between genetic and epigenetic alterations has been observed in cancer development, progression, and recurrence [32]. Both gene mutations and epigenetic alterations can be caused by exposure to various environmental factors, such as dietary components, smoke, and chemicals.

Epigenetic dysregulation, such as increased activity of histone deacetyltransferases (HDACs) and DNA methyltransferases (DNMTs) and changes in noncoding RNA expression, may lead to alterations in the transcription and expression of genes involved in the regulation of cell proliferation and differentiation, cell cycle, and apoptosis [32–37]. The present studies indicated that the level of HDAC5 expression was increased in human glioma and hepatocellular carcinoma, which promoted the proliferation of tumor cells via upregulating Six 1 (Sineoculis homeobox homolog 1) and Notch 1, respectively [38, 39]. A combination of HDAC and DNMT inhibitors contributed to cell cycle arrest in the G_2_/M phase and suppressed the growth of endometrial cancer through downregulation of Bcl-2 [37]. There are also multiple studies on miRNAs involved in regulating cell activities. For example, the upregulation of miR-96 and miR-153 promoted proliferation and colony formation of human prostate cancer cells [35, 36]. Evidence suggests that half of the tumor-suppressor genes are often inactivated via epigenetic, rather than genetic, mechanisms in sporadic cancers [23]. In addition, alterations in epigenetic processes mostly activate oncogenes, which enable cells to acquire cancer-promoting properties, such as uncontrolled proliferation, escape from apoptosis, and invasiveness. Accumulating evidence has suggested that targeting epigenetic modifications is a potent strategy for cancer prevention [23].

## 3. Epigenetic Mechanisms Underlying the Preventive Effects of SFN on Cancer

### 3.1. Histone Acetylation and Phosphorylation

Histone acetyltransferase (HAT) acetylates histones by adding acetyl groups to lysine residues in the N-terminal tail; this facilitates gene transcription by relaxing the chromatin structure to allow the transcription machinery to access the DNA. Conversely, HDACs repress transcription by removing acetyl groups. Many malignant neoplasms are characterized by increased expression and activity of HDACs. HDAC overexpression and overactivity are closely associated with transcriptional repression of the tumor-suppressor genes that are responsible for dysregulation of cell cycle, proliferation, differentiation, and apoptosis in malignances [23, 40, 41].

The food-based compound SFN, which is considered to be a HDAC inhibitor, has been shown to exert cancer preventive effects [22, 40, 41]. Treatment of various cancers, such as prostate [42], colon [43], and lung cancer [44], with SFN attenuated cell growth through inhibition of HDACs, accompanied by an increase in global or local histone acetylation. Moreover, SFN-mediated inhibition of HDACs contributed to reactivation of the tumor suppressor gene *p21* and the proapoptotic protein Bax. In the LnCaP and PC-3 prostate cancer cell lines, 15 *μ*M SFN treatment caused reexpression of p21^WAF1/CIP1^ due to reduced expression of class I and II HDACs and subsequent increases in acetylated histone H3 and H4 levels at the *p21^WAF1/CIP1^* promoter, which resulted in cell cycle arrest [42]. Interestingly, SFN also upregulated transcription of the *Bax* gene to induce apoptosis in prostate cancer cells by accelerating acetylation of histone H4 at the *Bax* promoter [45]. Similar changes in p21 and Bax reactivation, resulting from inhibition of HDACs and upregulation of acetylated histone H3 and H4, were observed in SFN-treated lung cancer cell lines and tumor tissues [44]. Ultimately, SFN with different concentrations (in vitro 15 *μ*M, in vivo 9 mM/mice/day) suppressed lung cancer growth *in vitro* and *in vivo* [44].

Additionally, HDACs can affect DNA damage and repair by altering the acetylation status of c-terminal-binding protein interacting protein (CtIP), a critical DNA repair protein [46]. In human colon cancer cells, coincident with inhibition of HDAC3 activity, SFN induced DNA damage and cell apoptosis via upregulation of CtIP acetylation and its subsequent degradation [43]. However, evidence for a direct interaction between HDACs and CtIP is lacking.

Inhibitory effects of SFN on HDACs were also observed *in vivo* [44, 47, 48]. In these studies, ingestion of SFN reduced the volume of prostate, breast, and lung tumors, accompanied by enhanced global histone acetylation and reduced HDAC activity [44, 47]. In human subjects, consumption of SFN-rich broccoli sprouts induced acetylation of histone H3 and H4, which was mainly attributed to inhibition of HDAC activity in circulating peripheral blood mononuclear cells (PBMCs) [48, 49].

The discrepancy in the concentration-effect relationship from in vitro to in vivo is a significant problem in the studies of natural phytochemicals, like SFN. To achieve the effective inhibition of HDAC activity, it was reported that the concentration of SFN used in vitro experiments was from 3 to 15 *μ*M, a single oral dose of 10 *μ*mol in mice, and 68 g broccoli sprouts in human [50]. An important factor determining the discrepancies is the conversion of glucosinolate to SFN by myrosinase-mediated hydrolysis. Isothiocyanate SFN was stored in broccoli sprouts as the precursor parent compound of glucosinolate, which was hydrolyzed to isothiocyanate by myrosinases released from the plants when raw vegetables were chopped, cut, or chewed, or by other myrosinase enzymes present in our gut [51, 52]. Therefore, mammalian tissues and cells in vitro cannot convert glucosinolate to SFN due to loss of endogenous myrosinase activity. However, glucosinolate is indeed converted to SFN by the myrosinases existing in gut microbial flora of animals and humans in vivo. Moreover, the bioavailability of SFN was about six times more than glucosinolates, which indicated the minimal conversion [53]. As an example, SFN at a concentration around 10 *μ*M effectively inhibited HDAC activity in mouse colonic mucosa in vivo, and humans would consume about 10^6^ g/day of broccoli sprouts to achieve similar plasma levels [54]. Altogether, the content of myrosinase in plants and variability of gut microbial flora are key factors of determining the discrepancies in the bioavailability of SFN from in vitro to in vivo.

In addition to acetylation modification, histones also undergo phosphorylation. A previous study demonstrated that increased phosphorylation of histone H1 is positively correlated with bladder cancer carcinogenesis and progression [55]. SFN reduced histone H1 phosphorylation by enhancing protein phosphatase 1*β* and 2A (PP1*β* and PP2A).

Collectively, these findings suggest that SFN may exert its anticancer effects through inhibition of HDACs and enhancement of phosphatases.

### 3.2. DNA Methylation

DNA methylation is an important epigenetic modification, mainly occurring within CPG islands in gene promoter regions. The establishment and maintenance of DNA methylation patterns requires the function of several DNA methyltransferases (DNMTs), which catalyze DNA methylation reactions, including DNMT1, which maintains methylation, and DNMT3a and DNMT3b, which catalyze de novo methylation [56]. Aberrant DNA methylation, such as promoter hypermethylation or hypomethylation, can lead to inactivation or activation of specific genes involved in tumorigenesis or progression, respectively. Aberrant DNA methylation is a reversible process and is often caused by the overexpression of DNMTs [57]. Therefore, DNMTs have become attractive targets for cancer chemoprevention.

Growing evidence indicates that SFN is a potential modulator of DNA methylation in cancer development and progression [22, 23, 40, 41]. As previously described, the expression levels of DNMTs, primarily DNMT1, 3a, and 3b, are decreased in SFN-treated breast, prostate, and cervical cancer cells [58–60]. Furthermore, the inhibitory effects of SFN on DNMTs can restore the expression and activation of silenced or repressed genes in cancer cells via promoter demethylation. Silencing of the cell cycle regulatory gene cyclin D2 by promoter hypermethylation was reported to be positively correlated with prostate cancer progression, and restoration of cyclin D2 expression induced cancer cell death [59]. An experiment with LNCap prostate cancer cells showed that SFN treatment reduced the expression of DNMT1 and 3b, resulting in a decrease in the global DNA methylation profile and cyclin D2 promoter methylation [59]. In addition, exposure of breast cancer cells to 10 *μ*M SFN reduced DNMT1 expression, which was accompanied by elevated expression of P21, the tumor suppressor phosphatase and tensin homologue (PTEN), and retinoic acid receptor beta 2 (RARbeta2) due to promoter demethylation [61]. Importantly, combining anticancer drugs, such as clofarabine (ClF) and withaferin A (WA), with SFN enhanced their anticancer effects, as reflected in the stronger growth arrest and apoptosis of cancer cells [61, 62]. Another study, using the same breast cancer cells, aimed at assessing the effects of SFN on human telomerase reverse transcriptase (hTERT), the catalytic regulatory subunit of telomerase. The results showed that SFN, at a dosage of 10 *μ*M, induced inhibition of DNMT1 and DNMT3a causing site-specific CpG demethylation in the first exon of the *hTERT* gene, thereby facilitating binding of the CTCF transcription repressor and hTERT repression [58]. This downregulation of hTERT expression promoted apoptosis in the breast cancer cells [58].

In the human cervical cancer cell line HeLa, SFN concentration significantly upregulated the expression of the tumor suppressor genes *RARβ*, *CDH1*, *DAPK1*, and *GSTP1*, as well as the expression of the proapoptosis protein Bax through inhibition of DNMT3b activity in a time-dependent manner, leading to the induction of cell cycle arrest and apoptosis [60].

The above results show that SFN functions as a cancer chemopreventive agent by modulating the expression of tumor-related genes through DNA methylation modification. Further studies in animal models of cancer are required to confirm and enhance the understanding of SFN on DNA methylation.

### 3.3. Regulation of Noncoding RNAs

A noncoding RNA (ncRNA) is an RNA molecule that functions without being translated into a protein. Abundant and functionally important ncRNAs include transfer RNAs and ribosomal RNAs, as well as small RNAs, such as microRNAs (miRNAs) and long ncRNAs (lncRNAs).

miRNAs are approximately 22 nucleotides in length and bind to complementary sites in the 3′-UTR of target messenger RNAs (mRNAs), leading to posttranscriptional repression or degradation [63]. miRNAs are negative regulators of target genes, and several miRNAs have been shown to be involved in the regulation of tumor cell proliferation, apoptosis, invasion, and metastasis. In addition, miRNA dysregulation has been shown to play an essential role in the development and progression of various cancers [64].

Several miRNAs, such as miR200c, miR-616-5p, and microRNA-21 (miR-21), have been shown to be targets of SFN in some human cancers [6, 65–67]. It is noteworthy that cancer stem cells (CSCs) are considered to be the driving force of carcinogenesis in oral squamous cell carcinoma (OSCC). In one study, miR200c targeting of Bmi1 was shown to be involved in the regulation of cancer stemness in OSCC-CSCs, including their self-renewal and tumor initiation properties [65]. SFN treatment (20 *μ*M) impaired cancer stemness by inducing the tumor-suppressive miRNA miR200c, which subsequently inhibited the migration, invasion, and clonogenicity of OSCC-CSCs in mouse models [65]. In addition to its effect on CSCs, SFN enhanced temozolomide-induced glioblastoma cell apoptosis [67] and reduced the viability and induced apoptosis of colon cancer cells [66] through downregulation of miR-21. In addition, SFN may specifically target miR616-5p to suppress the metastasis of non-small-cell lung cancer (NSCLC) cells [6]. Another study showed that miR-616-5p levels were increased in tissue samples of late-stage NSCLC, as well as three human NSCLC cell lines (H1299, 95C, and 95D) [6]. SFN downregulated miR-616-5p levels, which was accompanied by inactivation of the GSK3*β*/*β*-catenin pathway and inhibition of EMT to prevent NSCLC recurrence and metastasis [6].

lncRNAs are transcripts longer than 200 nucleotides that function as crucial regulators of gene transcription through their association with chromatin remodeling complexes [68]. Their aberrant expression endows cells with tumor initiation, high proliferation, and metastasis abilities [68]. Studies on the effects of SFN on lncRNAs are limited. However, a recent study showed that the lncRNA LINC01116 was upregulated in the human prostate cancer cell lines LNCaP and PC-3, and this upregulation was decreased by SFN (15 *μ*M), which was accompanied by inhibition of proliferation [69].

These studies suggest SFN as a promising chemopreventive agent and demonstrate that its anticancer effects partially involve epigenetic mechanisms, which are summarized in Table 1.

## 4. The Keap1/Nrf2 Antioxidant Pathway and Its Epigenetic Modification

### 4.1. The Keap1/Nrf2 Antioxidant Pathway and Cancer

**Figure 1:**
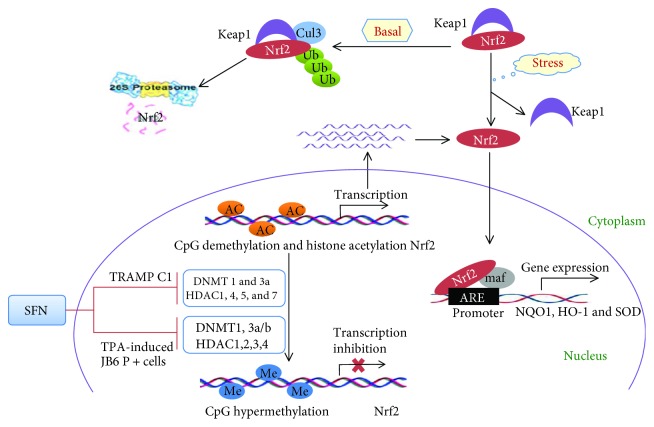
The Keap1/Nrf2 pathway and its epigenetic modification by SFN. Under basal conditions, *Keap1* binds to *Nrf2* in the cytoplasm, which promotes its proteasomal degradation via ubiquitination. Under oxidative stress, *Nrf2* dissociates from *Keap1* and then translocates into the nucleus and binds with the small protein Maf at ARE sequences in the promoter regions of target genes. This drives the expression of several cytoprotective genes, such as *HO-1*, *NQO1*, and *SOD*. In TRAMP C1 prostate cancer cells, SFN can inhibit the expression and activity of enzymes involved in epigenetic regulation, including DNMT1 and 3a, as well as HDAC1, 4, 5, and 7. Significant inhibition of DNMT1, DNMT3a/b, and HDAC1, 2, 3, and 4 has also been observed in TPA-induced mouse skin JB6 P_+_ cells treated by SFN, which reduces the CpG methylation and elevates histone acetylation of the *Nrf2* promoter. Ultimately, epigenetic regulation by SFN promotes the transcription of *Nrf2* and its subsequent nuclear translocation and activation.

Carcinogenesis is often associated with long-term exposure to oxidative stress resulting from the overproduction of high reactive oxygen species (ROS) and/or the impairment of the antioxidation system [70]. Nuclear factor erythroid-2- (NF-E2-) related factor 2 (Nrf2) is best known as a key transcription factor regulating the expression of antioxidant and detoxification genes, such as heme oxygenase-1 (*HO-1*), NAD(P)H:quinone oxidoreductase-1 (*NQO1*), and glutathione S-transferases (*GST*) [70]. A proposed model of Keap1-Nrf2 interaction is as follows: Under basal conditions, Nrf2 binds to its repressor Keap1 in the cytoplasm and subsequently undergoes proteasomal degradation via ubiquitination. Under oxidative stress, Nrf2 dissociates from Keap1 and then translocates to the nucleus. Intranuclear Nrf2 binds with the small protein Maf to antioxidant response element (ARE) sequences on target gene promoters, which drives the transcription of cytoprotective genes and provides protection against oxidative stress [71] (Figure 1).

Nrf2 has been traditionally regarded as a tumor suppressor. Low expression of cytoprotective genes, due to inactivation of Nrf2, has been shown to be related to tumor formation and progression. For example, Nrf2-deficient mice showed dramatically increased susceptibility to carcinogens and elevated lung metastasis, which was accompanied by increased ROS levels [72, 73]. The incidence, multiplicity, and size of colorectal tumors were increased in *Nrf2*-knockout mice [74]. Moreover, there are multiple studies describing the beneficial effects of Nrf2 activation in cancer chemoprevention [75]. These results suggest that activation of Nrf2 may be an important strategy in cancer prevention.

However, recent studies demonstrate that Nrf2 protects the survival of normal as well as cancer cells. The constitutive activation of Nrf2 creates an advantageous environment that favors the survival of malignant cells by preventing them from oxidative stress, chemotherapeutic drugs, and radiotherapy. This phenomenon has been called the “dark side of Nrf2.” The elevated Nrf2 expression induced by Keap1 mutation has been found in lung, gallbladder, liver [76], and prostate cancers [77], as well as malignant melanoma [78]. Moreover, the overexpression of Nrf2 contributed to clinical drug resistance and tumor growth, which was associated with poor prognosis of patients with cancer [77–80]. Additionally, inhibition of Nrf2 sensitized DU-145 prostate cancer cells to chemotherapeutic drugs, such as cisplatin and etoposide, and enhanced radiotherapy responsiveness [77]. These findings suggest that Nrf2 has a dual role in cancer development and therapy. Based on a body of studies, it seems that transient activation of Nrf2 in normal cells (where the Nrf2-Keap1 axis is intact) is protective; however, constitutive activation of Nrf2 (mutation of Keap1) promotes the survival and progression of malignant cells.

### 4.2. Epigenetic Modification of Nrf2 in Cancer Prevention

It has been shown that SFN induces Nrf2 to upregulate expression of its target genes, including antioxidant genes and phase II detoxification enzymes, to prevent carcinogenesis [14]. SFN not only modifies Keap1 cysteine residues, resulting in Nrf2 activation, but also restores Nrf2 expression through epigenetic mechanisms, including inhibition of DNMTs and HDACs [17, 20, 21]. In TRAMP C1 prostate cancer cells, it was reported that Nrf2 and its target gene *NQO1* were significantly decreased, resulting in extensive oxidative stress and DNA damage. SFN treatment upregulated the expression of Nrf2 and NQO1 by inhibiting DNMTs (DNMT1 and DNMT3a) and HDACs (HDAC1, HDAC4, HDAC5, and HDAC7) [20], which reduced the methylation level of CpGs and increased histone 3 acetylation at the *Nrf2* promoter. It was also observed that reactivation of Nrf2 and its target genes by SFN, via downregulation of CpG methylation at *Nrf2*, significantly inhibited TPA-induced JB6 P_+_ cellular transformation, with concomitant attenuation of the expression of DNMTs (DNMT1, DNMT3a, and DNMT3b) and HDACs (HDAC1, HDAC2, HDAC3, and HDAC4) [21] (Figure 1 and Table 1).

In addition, other natural phytochemicals of Nrf2 agonist, such as curcumin, 3,3′-diindolylmethane (DIM), Z-Ligustilide, apigenin, or Tanshinone IIA, have displayed the anticancer effect through epigenetic modification of Nrf2 [81–85]. For instance, curcumin, DIM, or Z-Ligustilide demethylated the CpGs in the *Nrf2* promoter and reactivated Nrf2 in the prostate of TRAMP mice and TRAMP C1 cells by the inhibition of DNMTs. Moreover, the hypermethylation of the *Nrf2* promoter could be reduced by apigenin or Tanshinone IIA in mouse skin epidermal JB6 P_+_ cells. Additionally, human prostate cancer cells treated with 5-aza/TSA (DNMT/HDAC inhibitor) restored the expression of Nrf2 [86]. These findings suggest that epigenetic restoration of Nrf2 may be an important strategy in cancer prevention.

## 5. The Clinical Studies and Future Perspectives

Human clinical studies have supported the chemopreventive effects of SFN on carcinogenesis. Firstly, several clinical trials evaluated the safety and tolerance of SFN at the doses employed. Two clinical phase I studies showed that broccoli sprout extracts containing SFN were well tolerated and caused no significant adverse effects (toxicities) when administered orally by healthy volunteers at a dose of 15 *μ*M for 7 days or women with breast cancer received 200 *μ*mol of on average 50 min prior to the surgery [87, 88]. A recent phase II clinical study on men with recurrent prostate cancer also confirms the safety of SFN [89]. In addition, another study assessed the clinical effectiveness of SFN in the patients with advanced pancreatic ductal adenocarcinoma (PDA). The data indicated that 90 mg/day of active SFN effectively inhibited tumor growth and increased the sensitivity of cancer cells to chemotherapeutics [90]. In human subjects, consumption of SFN-rich broccoli sprouts significantly inhibited HDAC activity in PBMCs [48, 49]. These clinical studies further suggest SFN as a promising anticancer agent and its potential epigenetic mechanisms.

Based on the above-mentioned studies, it is clear that the dietary compound SFN, which has little or no adverse side effects, exerts anticancer activities through multiple mechanisms, including epigenetic regulation. Thus, daily consumption of cruciferous vegetables rich in SFN is not only a healthy diet choice but also an effective chemopreventive strategy. SFN, as an inducer of Nrf2, shows the capacity to reactivate Nrf2 expression and its target cytoprotective genes to prevent carcinogenesis through epigenetic mechanisms, namely, CPG demethylation and histone acetylation of the Nrf2 promoter, via inhibition of DNMTs and HDACs. These studies have prompted us to propose epigenetic restoration of Nrf2 by SFN as an important strategy against oxidative damage-related diseases, including cancer, which may provide new research directions and preventive approaches for oxidative damage-related diseases.

## Figures and Tables

**Table 1:** The epigenetic regulation of sulforaphane (SFN) in cancer.

Epigenetic mechanisms	Cancer types	Epigenetic functions	Target genes/proteins	Anticancer effects	References
Histone acetylation	Prostate cancer cells (LnCaP and PC-3) and PC-3 cell xenografts	Inhibition of class I and II HDACs	Reactivation of p21 and Bax	Cell cycle arrest and apoptosis↑	[42, 45, 49]
	Colon cancer cells (HCT116)	Inhibition of HDAC3	CtIP: a critical DNA repair protein	DNA damage and apoptosis↑	[43]
			Acetylation of CtIP and its degradation		
	Lung cancer cells (A549 and H1299) and A549 cell xenografts	Inhibition of HDAC activity	Reactivation of p21 and Bax	Cell growth↓	[44]
				Apoptosis↑	

Histone phosphorylation	Bladder cancer cells (RT4, J82, and UMUC3) and UMUC3 cell xenografts	Inhibition of histone H1 phosphorylation	Increased PP1*β* and PP2A phosphatase	Carcinogenesis and progression↓	[55]

DNA methylation	Prostate cancer cells (LNCap)	Decreased expression of DNMT1 and 3b	Restoration of cyclin D2	Cancer cell death↑	[59]
	Human breast cancer cells (MCF-7 and MDA-MB-231)	Inhibition of DNMT1 expression	Restoration of P21, PTEN, and RARbeta2	Cell growth arrest and apoptosis↑	[61]
	Human breast cancer cells (MCF-7 and MDA-MB-231)	Decrease in DNMT1 and 3a expression and activity	Downregulation of hTERT expression	Apoptosis↑	[58]
	Cervical cancer cells (HeLa)	Inhibition of DNMT3b activity	Upregulation of RAR*β*, CDH1, DAPK1 and Bax	Cell cycle arrest and apoptosis↑	[60]

Noncoding RNA regulation	Oral squamous carcinoma cells (SAS and GNM); cancer stem cell xenografts (SAS and GNM)	Induction of miR-200c	Suppression of Bmi1	Cell migration, invasiveness, and growth↓	[65]
	95D and H1299 cells and in vivo xenografts	Downregulation of miR-616-5p	Inactivation of the GSK3*β*/*β*-catenin pathway	EMT and metastasis↓	[6]
	Human glioma cell lines (H4, SNB19, LN229, and U251) and colorectal cancer cells	Downregulation of miR 21	Inhibition of the Wnt/*β*-catenin pathway	Apoptosis↑	[66, 67]
				Cell viability↓	
	Prostate cancer cells (LNCaP and PC-3)	Decreased expression of the lncRNA LINC01116		Cell proliferation↓	[69]

CPG demethylation and histone acetylation at the *Nrf2* promoter	Mouse skin epidermal JB6 (JB6 P_+_) cells and prostate cancer (TRAMP C1) cells	Inhibition of DNMT1, 3a, and 3b and HDAC1–5 and HDAC7	The reactivation of Nrf2	Cell transformation and development↓	[20, 21]
